# Suicide risk in prostate cancer patients: epidemiological trends and a predictive modeling

**DOI:** 10.1186/s12888-026-07806-7

**Published:** 2026-01-19

**Authors:** Yu-Xuan Yang, Gui-Chen Ye, Qing-Xu Yao, Xing-Yu Zhong, Yi-Fan Xiong, Ming-Liang Zhong, Xi Gong, Shao-Gang Wang, Qi-Dong Xia

**Affiliations:** Department and Institute of Urology, Tongji Hospital, Tongji Medical College, Huazhong University of Science and Technology, No. 1095 Jiefang Avenue, Wuhan, 430030 P. R. China

**Keywords:** Suicide, Prostate cancer, Nomogram, Prognosis

## Abstract

**Background:**

Prostate cancer (PCa) is a common malignancy among men. Although survival rates have steadily improved, some patients still face an increased risk of suicide following diagnosis. Understanding the temporal patterns and key risk factors associated with suicide after a PCa diagnosis is crucial for developing effective intervention strategies. However, comprehensive models for individualized suicide risk prediction remain limited.

**Methods:**

This study utilized data from the SEER database covering the period from 2010 to 2021. First, the suicide mortality trend among PCa patients was assessed by calculating the standardized mortality ratio (SMR). Subsequently, variable selection was performed using the least absolute shrinkage and selection operator (LASSO) regression, combined with Cox proportional hazards regression to further identify independent risk factors associated with suicide risk. A total of 404,120 eligible patients were included for model construction and randomly divided into training and validation cohorts at a 7:3 ratio. Based on the results of multivariable Cox regression, a nomogram prediction model was developed. The model’s performance was evaluated and validated using the concordance index (C-index), receiver operating characteristic (ROC) curves, and calibration curves.

**Result:**

Among 475,139 PCa patients identified, the suicide risk was highest during the first six months after diagnosis (SMR = 26.007, 95% CI: 19.303–34.287) and declined significantly after five years. Nine independent risk factors, including age, race, and Gleason score, were incorporated into the nomogram model. The model showed strong predictive performance, with C-indices of 0.714 and 0.685 in the training and validation cohorts, respectively. ROC curve analysis demonstrated good discrimination ability, and calibration curves indicated excellent agreement between predicted and observed outcomes.

**Conclusion:**

This study highlights the heightened risk of suicide among PCa patients, particularly within the first six months following diagnosis, underscoring the urgent need for early psychological intervention. The developed nomogram provides an effective tool for personalized suicide risk assessment, enabling clinicians to identify high-risk patients and implement targeted preventive strategies. Integrating mental health care into routine oncology practice may significantly improve overall patient outcomes.

**Clinical trial number:**

Not applicable.

**Supplementary Information:**

The online version contains supplementary material available at 10.1186/s12888-026-07806-7.

## Introduction

Prostate cancer is the second most prevalent malignancy globally and the fifth leading cause of cancer-related mortality in males [1].Researchers have extensively investigated factors influencing cancer mortality to develop more effective intervention strategies. A substantial proportion of PCa patients succumb not to the malignancy itself, but to non-cancer causes, particularly cardiovascular disease, Alzheimer’s disease, and chronic obstructive pulmonary disease (COPD) [2, 3]. Beyond these competing risks of mortality, patients also face significant psychosocial challenges that can lead to severe mental health outcomes, including suicide.

The American Society of Clinical Oncology (ASCO) identifies psychological distress as a significant clinical manifestation in cancer patients [4], with suicidal behavior representing an extreme outcome of these psychological conditions. Globally, approximately 800,000 individuals die by suicide each year, accounting for 1.5% of all deaths [5]. A cancer diagnosis can provoke profound emotional turmoil, as patients grapple with uncertainty, fear of disease progression, and concerns about the burden on their families, all of which exacerbate psychological distress [6, 7]. In addition, a poor prognosis is also a key factor that can overwhelm patients’ psychological defenses and lead to suicide. For example, highly aggressive cancers such as pancreatic cancer and small cell lung cancer are associated with particularly high suicide risks [8], with pancreatic cancer having the highest suicide rate among male patients [9, 10]. However, even prostate cancer, which has a five-year relative survival rate of 97% and is considered less aggressive [11], still exhibits a high suicide rate of 25.4 per 100,000 person-years [12], indicating that prostate cancer patients face unique psychological challenges. Owing to its association with male identity, sexual dysfunction, and post-treatment urinary incontinence, prostate cancer imposes an additional psychological burden, further intensifying patients’ mental health struggles [13]. Notably, PCa patients face a significantly heightened suicide risk within the first year following diagnosis (RR = 2.01, 95% CI: 1.52–2.64). Particularly among elderly patients aged ≥ 75, the suicide risk is more pronounced within 12 months after diagnosis [14], the high vulnerability of this population underscores the importance of early psychological assessment and intervention. Additionally, hormone therapy, a common treatment for advanced PCa, has been proven to be associated with depression and emotional instability [15], further emphasizing the necessity of comprehensive psychological care.

While prior research has extensively examined PCa recurrence, overall survival (OS), and cancer-specific survival (CSS) [16], suicide risk in this population remains an underrecognized yet critical issue. Existing studies have identified several risk factors for suicide among PCa patients, including high-risk disease status, lack of radical prostatectomy, and poor social support networks [17]. However, most of these studies are limited by small sample sizes or short follow-up durations, restricting their generalizability. Large-scale, population-based studies focusing on long-term suicide risk assessment remain scarce, leaving critical gaps in our understanding of suicide epidemiology among PCa patients.

Given the significant psychological burden of PCa and the heightened suicide risk, a systematic approach to early risk identification is urgently needed. Current clinical practice lacks effective tools to quantify suicide risk, making it challenging to implement timely interventions. To address this gap, our study utilizes a large and diverse cohort of 475,139 PCa patients from the SEER database. The size and heterogeneity of this cohort overcome the limitations of previous small-sample studies, providing a robust foundation for developing and validating a nomogram-based prediction model with potential clinical applicability. By integrating multiple risk factors, this study aims to provide an individualized risk assessment tool to support future suicide prevention strategies and improve timely mental health interventions for high-risk patients.

## Materials and methods

### The study population and patient selection

This study is based on the SEER 17 registries, which covers approximately 30% of the U.S. population and includes 17 population-based cancer registries, providing survival data, clinical pathology information, and demographic data. This study used SEER*Stat software to extract data on PCa patients diagnosed from 2010 to 2021, identified using the research center code C61.9. Additionally, suicide rate data for the general U.S. population (reference group) was obtained from the Centers for Disease Control and Prevention (CDC) web-based Fatal Injury and Violence Reports (https://wisqars.cdc.gov/nvdrs). Since both the SEER database and CDC data are publicly available, this study does not require additional patient informed consent.

### Variable collection and outcome definition

**Fig. 1:**
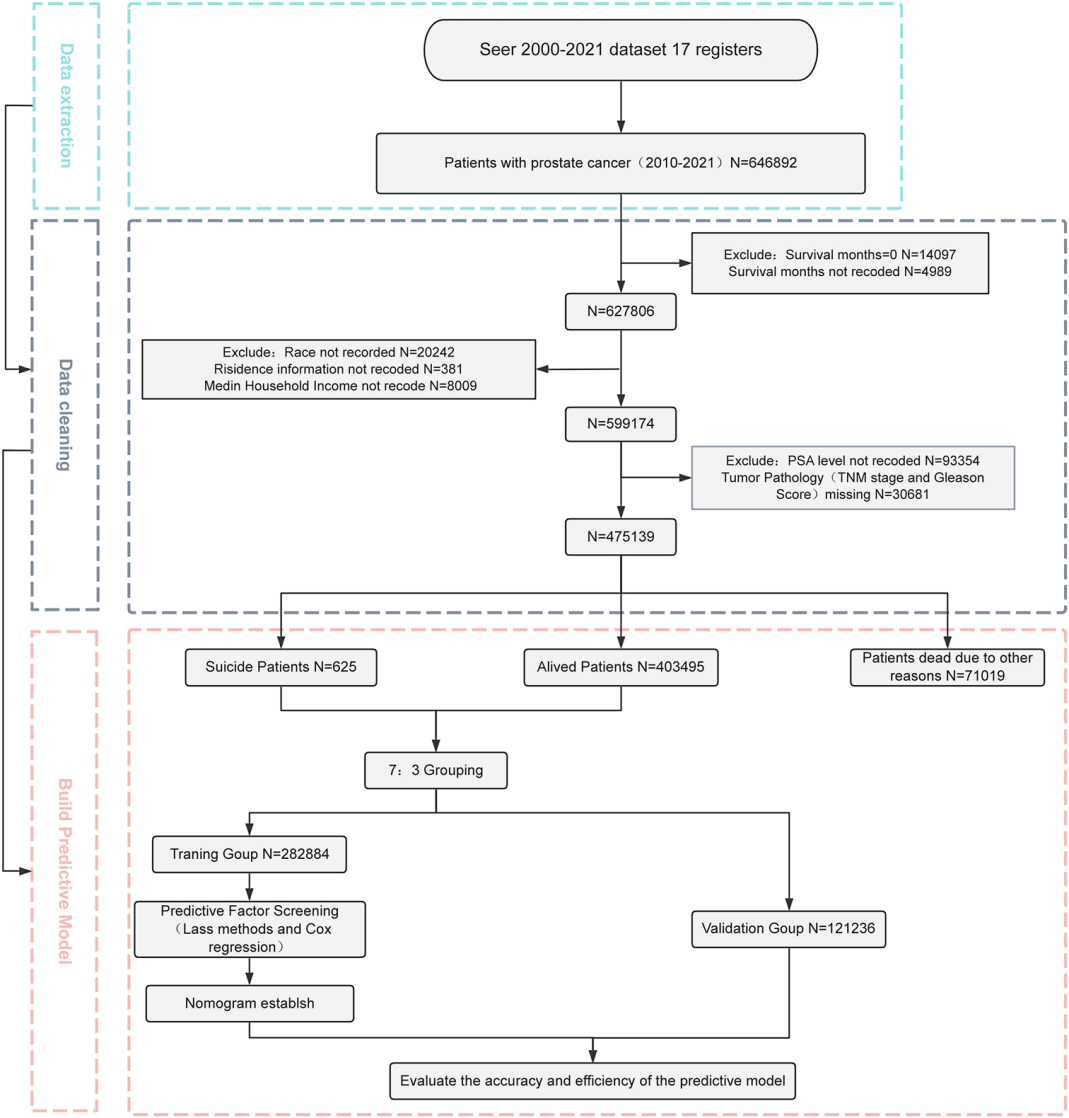
Flowchart of the patients screening process

This study included PCa patients diagnosed between 2010 and 2021 from the SEER database, with a focus on cases with complete follow-up data. The primary endpoint of the study was suicide-related mortality, defined as “suicide and self-inflicted injury” according to the SEER database’s “COD to site record” classification. Patient survival time was calculated from the diagnosis date to death or the end of the follow-up period. The independent variables included demographic characteristics (age, race, marital status), socioeconomic factors (residence, income level), tumor-related indicators (TNM stage, Gleason score, PSA level), treatment methods (surgery, radiation therapy, endocrine therapy), and follow-up information. Gleason score is a well-established pathological grading system for prostate cancer, assessing tumor tissue morphology on a scale from 2 to 10. Higher scores indicate poorer differentiation and greater aggressiveness, serving as an important indicator for prognosis evaluation and treatment guidance [18, 19]. Prostate-specific antigen (PSA), secreted by prostate epithelial cells, is a commonly used serum biomarker for prostate cancer screening, diagnosis, and disease monitoring [20]. For ease of analysis, some continuous variables were converted into categorical variables. Age was categorized as < 60 years, 60–69 years, 70–79 years, and ≥ 80 years, while PSA levels were categorized as < = 4 ng/mL, 4–10 ng/mL, 10–20 ng/mL, and > 20 ng/mL [21]. Since some patients died from causes other than PCa, overall mortality data were also recorded and adjusted for in subsequent analyses. A total of 475,139 PCa patients were included after excluding those with incomplete data. A detailed screening process is shown in Fig. 1.

### Statistical analysis

The dataset was split into training and validation sets with a 7:3 ratio. Initially, correlation coefficient analysis was conducted to select variables with weak correlation, ensuring that variables in subsequent analyses were not highly correlated with each other. Categorical variables were presented as frequencies (%), while continuous variables were shown as means ± standard deviations (SD). Group comparisons were conducted using the chi-square test for categorical variables and Student’ s t-test for continuous variables. Variable selection was performed using LASSO regression, and the selected variables were further included in the multivariable Cox regression model. Key predictive factors were determined through stepwise regression (*P* < 0.05), and a nomogram was constructed for individualized risk prediction. In addition, we conducted Fine–Gray analyses in the overall cohort to validate the robustness of the identified risk factors and plotted cumulative incidence function (CIF) curves stratified by relevant covariates to visualize the cumulative risk of suicide. Model performance was assessed using the ROC curves C-index to assess the discrimination ability, and calibration curves were used to validate the prediction accuracy. Kaplan-Meier survival analysis was conducted to evaluate the suicide survival probability in different risk groups, and the Log-rank test was used to compare group differences. Additionally, the AUC of the univariate ROC curve was calculated and compared with the AUC of the multivariable model to assess the independent predictive value of variables. All statistical analyses were performed in R 4.2.1, and *P* < 0.05 was considered statistically significant.

## Result

### Baseline characteristics and cause of death rankings of PCa patients

This study utilized SEER database data from 2010 to 2021, encompassing 475,139 PCa patients. Among them, 79.3% were White, the largest age group was 60–69 years (41.2%), and 68.5% were married. To explore the factors related to suicide, patients were categorized into three groups: suicide death (625 patients, 0.13%), death from other causes (71,019 patients, 14.95%), and survival (403,495 patients, 84.92%). The median age at diagnosis was 68 years, 66 years in the suicide group, and 72 years in the other cause death group. Additional baseline characteristics, including PSA levels, tumor stage, and treatment modalities, are provided in Table 1.

**Table 1:** Baseline characteristics of prostate cancer patients (2010–2021)

	Overall	Alive	Suicide	Other reasons for death	*P* Value
	(*N* = 475139)	(*N* = 403495)	(*N* = 625)	(*N* = 71019)	
**Diagnosis Year**					< 0.001
2010–2013	155,680 (32.8%)	114,311 (28.3%)	361 (57.8%)	41,008 (57.7%)	
2014–2017	147,931 (31.1%)	125,510 (31.1%)	201 (32.2%)	22,220 (31.3%)	
2018–2021	171,528 (36.1%)	163,674 (40.6%)	63 (10.1%)	7791 (11.0%)	
**Age (Mean [SD])**	66.1 (8.49)	65.3 (8.08)	66.4 (9.41)	71.0 (9.08)	< 0.001
**Age (%)**					< 0.001
<60	104,819 (22.1%)	97,084 (24.1%)	165 (26.4%)	7570 (10.7%)	
>=80	210,807 (44.4%)	186,981 (46.3%)	231 (37.0%)	23,595 (33.2%)	
60–69	130,758 (27.5%)	103,910 (25.8%)	176 (28.2%)	26,672 (37.6%)	
70–79	28,755 (6.1%)	15,520 (3.8%)	53 (8.5%)	13,182 (18.6%)	
**Race (%)**					< 0.001
Black	75,908 (16.0%)	63,461 (15.7%)	38 (6.1%)	12,409 (17.5%)	
Other	28,644 (6.0%)	24,882 (6.2%)	12 (1.9%)	3750 (5.3%)	
White	370,587 (78.0%)	315,152 (78.1%)	575 (92.0%)	54,860 (77.2%)	
**PSA Level (%)**					< 0.001
<=4	48,241 (10.2%)	42,670 (10.6%)	60 (9.6%)	5511 (7.8%)	
>20	284,118 (59.8%)	253,646 (62.9%)	348 (55.7%)	30,124 (42.4%)	
10–20	80,576 (17.0%)	66,346 (16.4%)	120 (19.2%)	14,110 (19.9%)	
4–10	62,204 (13.1%)	40,833 (10.1%)	97 (15.5%)	21,274 (30.0%)	
**Gleason Score (%)**					< 0.001
Gleason Score < = 6	166,974 (35.1%)	149,351 (37.0%)	210 (33.6%)	17,413 (24.5%)	
Gleason Score = 7	199,262 (41.9%)	175,037 (43.4%)	248 (39.7%)	23,977 (33.8%)	
Gleason Score > = 8	108,903 (22.9%)	79,107 (19.6%)	167 (26.7%)	29,629 (41.7%)	
**T Stage (%)**					< 0.001
T0 + TX	7153 (1.5%)	4705 (1.2%)	13 (2.1%)	2435 (3.4%)	
T1	205,849 (43.3%)	171,949 (42.6%)	283 (45.3%)	33,617 (47.3%)	
T2	192,199 (40.5%)	167,342 (41.5%)	240 (38.4%)	24,617 (34.7%)	
T3	64,257 (13.5%)	56,375 (14.0%)	79 (12.6%)	7803 (11.0%)	
T4	5681 (1.2%)	3124 (0.8%)	10 (1.6%)	2547 (3.6%)	
**N Stage (%)**					< 0.001
N0 + NX	453,496 (95.4%)	388,278 (96.2%)	602 (96.3%)	64,616 (91.0%)	
N1	21,643 (4.6%)	15,217 (3.8%)	23 (3.7%)	6403 (9.0%)	
**M Stage (%)**					< 0.001
M0	451,296 (95.0%)	392,389 (97.2%)	587 (93.9%)	58,320 (82.1%)	
M1	23,843 (5.0%)	11,106 (2.8%)	38 (6.1%)	12,699 (17.9%)	
**Chemotherapy (%)**					< 0.001
No/Unknown	469,612 (98.8%)	400,475 (99.3%)	617 (98.7%)	68,520 (96.5%)	
Yes	5527 (1.2%)	3020 (0.7%)	8 (1.3%)	2499 (3.5%)	
**Radiotherapy (%)**					< 0.001
No/Unknown	304,575 (64.1%)	261,070 (64.7%)	402 (64.3%)	43,103 (60.7%)	
Yes	170,564 (35.9%)	142,425 (35.3%)	223 (35.7%)	27,916 (39.3%)	
**Marital Status (%)**					< 0.001
Married	316,559 (66.6%)	275,577 (68.3%)	323 (51.7%)	40,659 (57.3%)	
Other	104,965 (22.1%)	83,259 (20.6%)	207 (33.1%)	21,499 (30.3%)	
Single	53,615 (11.3%)	44,659 (11.1%)	95 (15.2%)	8861 (12.5%)	
**Residence (%)**					< 0.001
Metropolitan	418,803 (88.1%)	357,902 (88.7%)	517 (82.7%)	60,384 (85.0%)	
Non-metropolitan	56,336 (11.9%)	45,593 (11.3%)	108 (17.3%)	10,635 (15.0%)	
**Median Household Income**,** $ (%)**					< 0.001
<45,000$	15,895 (3.3%)	12,046 (3.0%)	23 (3.7%)	3826 (5.4%)	
>=75,000$	265,513 (55.9%)	233,875 (58.0%)	273 (43.7%)	31,365 (44.2%)	
45,000$-74,999$	193,731 (40.8%)	157,574 (39.1%)	329 (52.6%)	35,828 (50.4%)	
**Surgical Treatment (%)**					< 0.001
None	293,816 (61.8%)	239,232 (59.3%)	411 (65.8%)	54,173 (76.3%)	
Other	23,570 (5.0%)	16,590 (4.1%)	38 (6.1%)	6942 (9.8%)	
RP	157,753 (33.2%)	147,673 (36.6%)	176 (28.2%)	9904 (13.9%)	

Continuous data are shown as mean (SD) and categorical data are shown as percentage

**Fig. 2:**
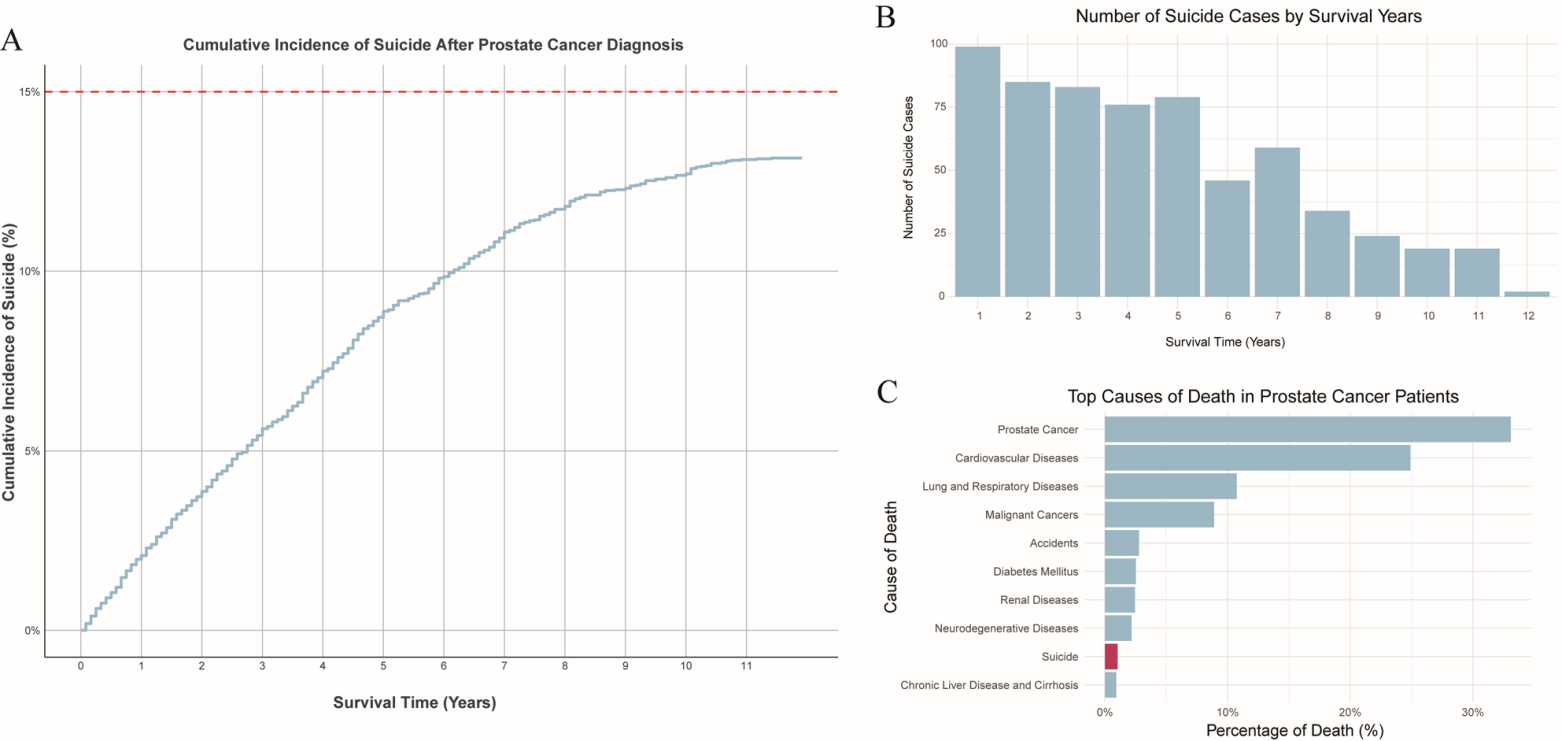
Suicide Risk and Causes of Death in Prostate Cancer Patients. (**A**) Cumulative incidence of suicide among prostate cancer patients over time; (**B**) Annual occurrences of suicide incidents post-PCa diagnosis; (**C**) Leading causes of death in prostate cancer patients

To better understand the factors contributing to mortality in PCa patients, we summarized and ranked the causes of death within the cohort (Fig. 2C). The findings revealed that prostate cancer-specific deaths had the highest proportion, followed by cardiovascular diseases. Suicide ranked ninth among the causes of death in PCa patients, accounting for 0.87% of all death cases, underscoring the need for predictive models to assess suicide risk in these patients.

### Suicide rates and SMR in PCa patients

This study included 625 PCa patients who died by suicide, with an overall suicide rate of 25.4 per 100,000 person-years (SMR = 1.198, 95% CI: 1.107–1.297). In terms of time trends, the suicide rate was highest between 2010 and 2013 (26.8 per 100,000 person-years, SMR = 1.321), followed by a slight decline, reaching 19.6 per 100,000 person-years (SMR = 0.89) during 2018–2021. Among different age groups, the suicide rate increased with age, with the highest rate observed in patients aged 80 and above (49.6 per 100,000 person-years, SMR = 1.182). Racial analysis showed that white patients had the highest suicide rate (29.7 per 100,000 person-years, SMR = 1.243). Additionally, patients with Gleason score ≥ 8, PSA > 20 ng/mL, T4 stage, and M1 stage exhibited significantly higher suicide rates and SMRs. Treatment modality and social factors also influenced suicide risk: patients who did not undergo radical surgery (28.6 per 100,000 person-years, SMR = 1.345), single patients (37.1 per 100,000 person-years, SMR = 1.748), or those living in non-metropolitan areas (38.0 per 100,000 person-years, SMR = 1.691) had higher suicide rates. The detailed information can be found in Table S1. These findings suggested that high-risk PCa patients and those with limited social support may require more mental health interventions and support.

Further analysis of changes in suicide rates during the follow-up period after diagnosis (Table 2) revealed that the suicide risk peaked within the first 6 months after diagnosis (SMR = 26.007, 95% CI: 19.303–34.287), followed by a gradual decline. Suicide risk remained significantly elevated during the 0.5-1 year (SMR = 9.895) and 1–2 year (SMR = 5.194) periods. By 3–5 years, suicide risk decreased to 2.023, and after 5 years, the suicide risk was even lower than that of the general population (SMR = 0.515, 95% CI: 0.446–0.591). Figure 2B shows that the peak of suicide risk occurred mainly in the early stages following diagnosis, emphasizing the importance of psychological support during this period.

**Table 2:** Variations in suicide SMRs per 100,000 person-years at risk during follow-up after prostate cancer diagnosis

Follow-up time from diagnosis	Observed No. of suicides	Person-years	Expected-suicides	SMR (95% CI)
0-0.5 year	50	9051.667	1.923	26.007 (19.303–34.287)
0.5-1 year	49	23315.583	4.952	9.895 (7.32–13.081)
1–2 years	85	77055.417	16.367	5.194 (4.148–6.422)
2–3 years	83	131523.833	27.936	2.971 (2.366–3.683)
3–5 years	155	360784.833	76.631	2.023 (1.717–2.367)
more than 5 years	203	1856682.75	394.359	0.515 (0.446–0.591)

### Risk factors for suicide in PCa patients

To further investigate the risk factors for suicide in PCa patients, we conducted a comprehensive analysis comparing suicide patients with surviving patients, including a total of 404,210 PCa patients. The cohort was then divided into a validation set (282,884 cases) and a training set (121,236 cases) in a 7:3 ratio, with balanced baseline characteristics between the two groups (*P* > 0.05). The average age of the patients was 65.3 years, and the distribution of diagnosis years was uniform. The majority of patients were White (78.1%), and most had PSA levels concentrated in the range of 4–10 ng/mL (62.9%). Detailed clinical baseline characteristics are presented in Table 3.

**Table 3:** The baseline characteristics table of patients in the training and validation sets

	Overall (*N* = 404120)	Training Set (*N* = 282884)	Validation Set (*N* = 121236)	*P* Value
**Diagnosis Year (%)**				0.4012
2010–2013	114,672 (28.4%)	80,439 (28.4%)	34,233 (28.2%)	
2014–2017	125,711 (31.1%)	87,872 (31.1%)	37,839 (31.2%)	
2018–2021 **Age (Mean [SD])**	163,737 (40.5%) 65.3 (8.09)	114,573 (40.5%) 65.3 (8.08)	49,164 (40.6%) 65.3 (8.09)	0.2936
**Age at diagnosis (%)**				0.4809
<60	97,249 (24.1%)	68,191 (24.1%)	29,058 (24.0%)	
>=80	15,573 (3.9%)	10,830 (3.8%)	4743 (3.9%)	
60–69	187,212 (46.3%)	131,075 (46.3%)	56,137 (46.3%)	
70–79	104,086 (25.8%)	72,788 (25.7%)	31,298 (25.8%)	
**Race (%)**				0.3896
Black	63,499 (15.7%)	44,529 (15.7%)	18,970 (15.6%)	
Other	24,894 (6.2%)	17,340 (6.1%)	7554 (6.2%)	
White	315,727 (78.1%)	221,015 (78.1%)	94,712 (78.1%)	
**PSA Level (%)**				0.7286
<=4	42,730 (10.6%)	29,951 (10.6%)	12,779 (10.5%)	
>20	40,930 (10.1%)	28,711 (10.1%)	12,219 (10.1%)	
10–20	66,466 (16.4%)	46,427 (16.4%)	20,039 (16.5%)	
4–10	253,994 (62.9%)	177,795 (62.9%)	76,199 (62.9%)	
**Gleason Score (%)**				0.5134
Gleason Score < = 6	149,561 (37.0%)	104,570 (37.0%)	44,991 (37.1%)	
Gleason Score = 7	175,285 (43.4%)	122,865 (43.4%)	52,420 (43.2%)	
Gleason Score > = 8	79,274 (19.6%)	55,449 (19.6%)	23,825 (19.7%)	
**T Stage (%)**				0.3899
T0 + TX	4718 (1.2%)	3307 (1.2%)	1411 (1.2%)	
T1	172,232 (42.6%)	120,323 (42.5%)	51,909 (42.8%)	
T2	167,582 (41.5%)	117,419 (41.5%)	50,163 (41.4%)	
T3	56,454 (14.0%)	39,659 (14.0%)	16,795 (13.9%)	
T4	3134 (0.8%)	2176 (0.8%)	958 (0.8%)	
**N Stage (%)**				0.5764
N0 + NX	388,880 (96.2%)	272,247 (96.2%)	116,633 (96.2%)	
N1	15,240 (3.8%)	10,637 (3.8%)	4603 (3.8%)	
**M Stage (%)**				0.8866
M0	392,976 (97.2%)	275,090 (97.2%)	117,886 (97.2%)	
M1	11,144 (2.8%)	7794 (2.8%)	3350 (2.8%)	
**Chemotherapy (%)**				0.8923
No/Unknown	401,092 (99.3%)	280,761 (99.2%)	120,331 (99.3%)	
Yes	3028 (0.7%)	2123 (0.8%)	905 (0.7%)	
**Radiotherapy (%)**				0.5069
No/Unknown	261,472 (64.7%)	182,938 (64.7%)	78,534 (64.8%)	
Yes	142,648 (35.3%)	99,946 (35.3%)	42,702 (35.2%)	
**Cause of Death (%)**				0.5124
Alive	403,495 (99.8%)	282,439 (99.8%)	121,056 (99.9%)	
Suicide	625 (0.2%)	445 (0.2%)	180 (0.1%)	
**Marital Status (%)**				0.9310
Married	275,900 (68.3%)	193,181 (68.3%)	82,719 (68.2%)	
Other	83,466 (20.7%)	58,390 (20.6%)	25,076 (20.7%)	
Single	44,754 (11.1%)	31,313 (11.1%)	13,441 (11.1%)	
**Residence (%)**				0.6780
Metropolitan	358,419 (88.7%)	250,855 (88.7%)	107,564 (88.7%)	
Non-metropolitan	45,701 (11.3%)	32,029 (11.3%)	13,672 (11.3%)	
**Median Household Income**,** $ (%)**				0.5934
<45,000$	12,069 (3.0%)	8412 (3.0%)	3657 (3.0%)	
>=75,000$	234,148 (57.9%)	163,825 (57.9%)	70,323 (58.0%)	
45,000$-74,999$	157,903 (39.1%)	110,647 (39.1%)	47,256 (39.0%)	
**Surgical Treatment (%)**				0.1966
None	239,643 (59.3%)	167,623 (59.3%)	72,020 (59.4%)	
Other	16,628 (4.1%)	11,542 (4.1%)	5086 (4.2%)	
RP	147,849 (36.6%)	103,719 (36.7%)	44,130 (36.4%)	

Continuous data are shown as mean (SD) and categorical data are shown as percentage

**Fig. 3:**
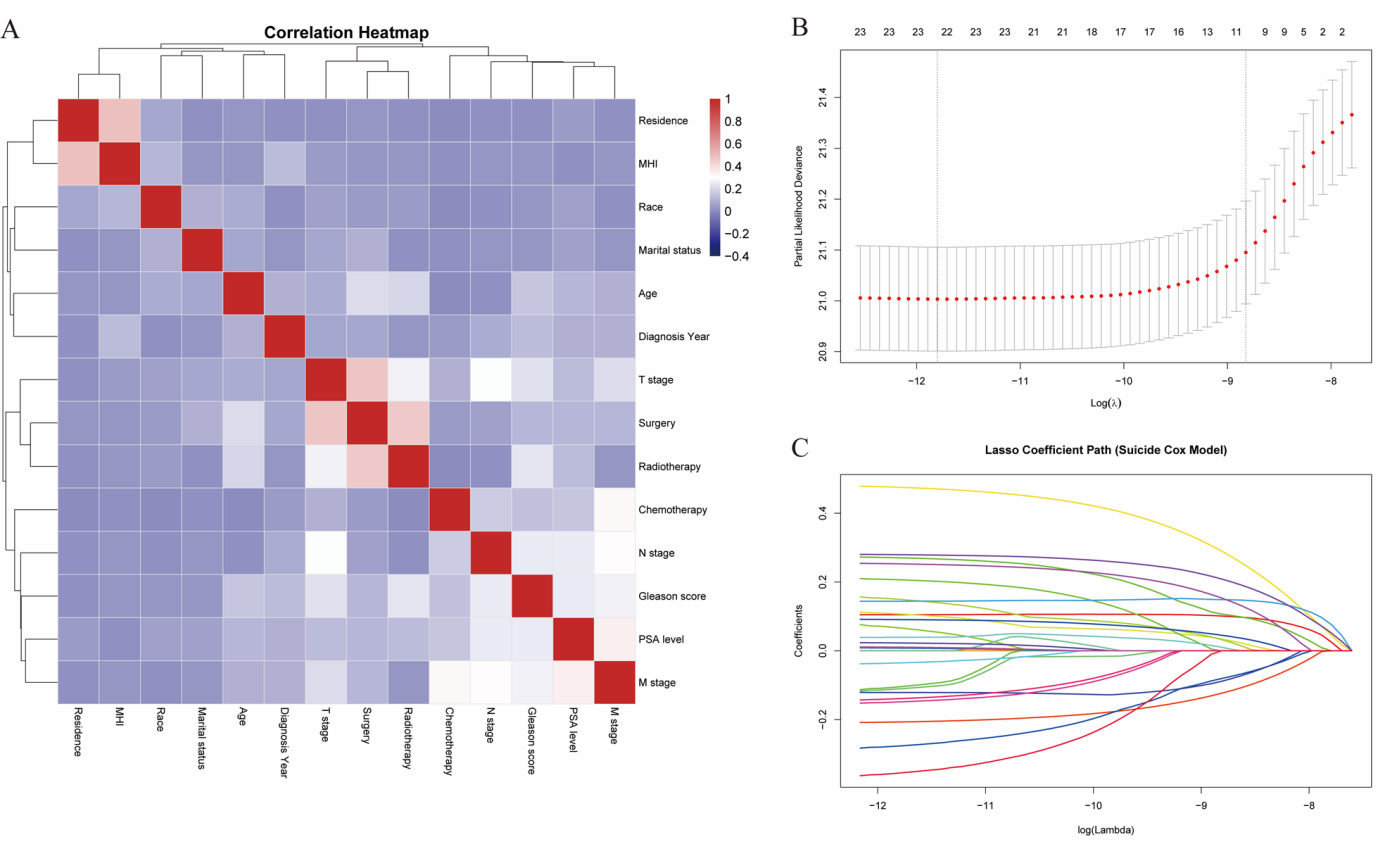
Correlation Analysis and LASSO Regression Results. (**A**) Correlation heatmap illustrating the relationships among key variables; (**B**) Determination of the optimal λ parameter through cross-validation in the Lasso model; (**C**) Changes in variable coefficients during the Lasso regression process

**Fig. 4:**
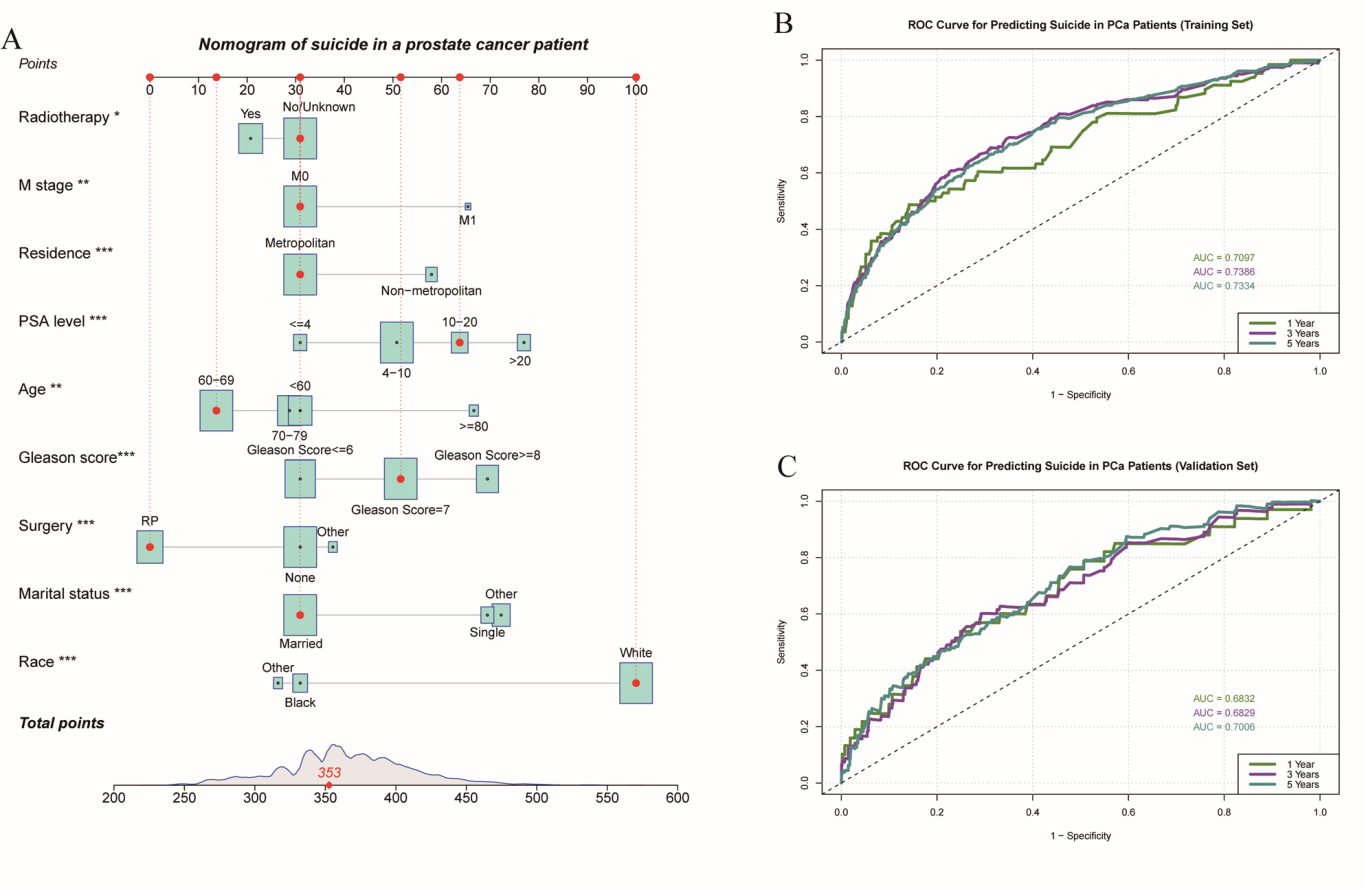
Development and validation of the survival prediction nomogram: (**A**) Nomogram for predicting suicide at different time points based on nine risk factors; (**B**–**C**) Receiver operating characteristic (ROC) curves illustrating the predictive performance of the nomogram in the training and validation sets at 1 year, 3 years, and 5 years, respectively. The area under the curve (AUC) values indicate the model’s discriminatory ability

In the correlation analysis of variables, the results showed no significant collinearity between the factors (Fig. 3A). Subsequently, we used LASSO regression to select key risk factors and incorporated them into a multivariable Cox regression analysis to identify independent prognostic factors (*P* < 0.05) (Table S2). The analysis revealed that age ≥ 80 years (HR = 1.88, 95% CI: 1.27–2.78), White race (HR = 3.49, 95% CI: 2.36–5.16), Gleason score ≥ 8 (HR = 1.93, 95% CI: 1.44–2.59), M1 stage (HR = 1.77, 95% CI: 1.12–2.79), PSA levels of 10–20 ng/mL (HR = 1.74, 95% CI: 1.15–2.61) or > 20 ng/mL (HR = 2.15, 95% CI: 1.38–3.33), unmarried status (HR = 1.93, 95% CI: 1.45–2.57) and living in non-urban areas (HR = 1.38, 95% CI: 1.04–1.82) were all significantly associated with a higher suicide risk. In contrast, radical prostatectomy (HR = 0.57, 95% CI: 0.42–0.78) and radiotherapy (HR = 0.84, 95% CI: 0.67–0.94) were protective factors, suggesting that active treatment might improve the suicide survival prognosis for patients. Based on these key variables, we developed a survival prediction model and visually presented individualized predictions through a nomogram (Fig. 4A), providing a reliable tool for clinically assessing suicide risk in PCa patients. Furthermore, the model was assessed and found to satisfy the proportional hazards assumption, which further enhances the reliability of its predictions (Figure S2).

To further validate the robustness of these identified risk factors, we performed Fine–Gray analyses in the overall cohort, treating non-suicide deaths as competing events. In this context (Table S2), radiotherapy (sHR = 0.83, 95% CI: 0.69–1.21) was no longer statistically significant. Nevertheless, the remaining risk factors, including White race, Gleason score ≥ 8, M1 stage, PSA levels > 20 ng/mL, unmarried status, and residence in non-metropolitan, remained robustly associated with higher suicide risk. Subsequently, CIF curves were plotted for these key risk factors (Figure S1), providing a visual representation of the stratified cumulative suicide risk over time.

### Validation and evaluation of suicide prediction model in PCa patients

**Fig. 5:**
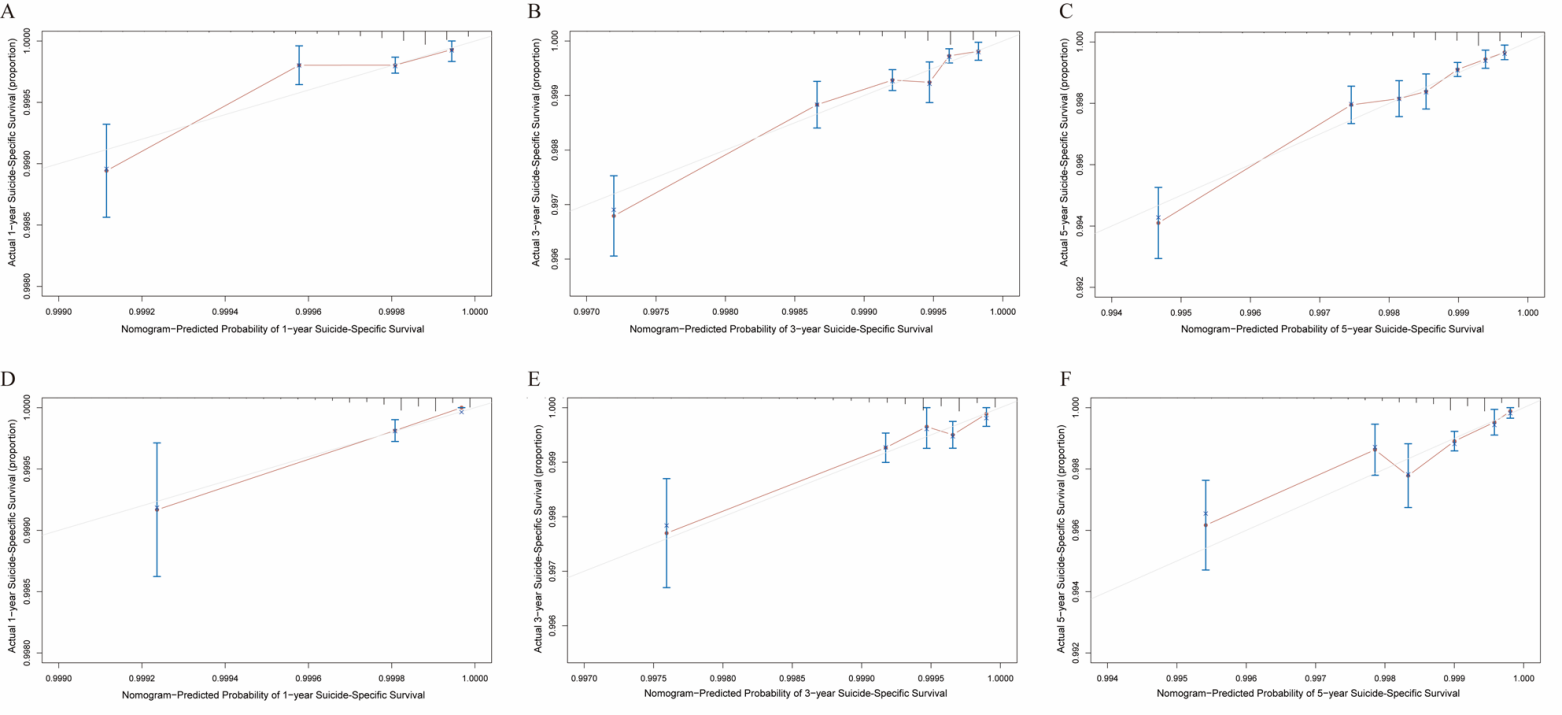
Calibration curves for the training set and validation set predicting suicide in prostate cancer patients at 1 year (**A**, **D**), 3 years (**B**, **E**), and 5 years (**C**, **F**)

**Fig. 6:**
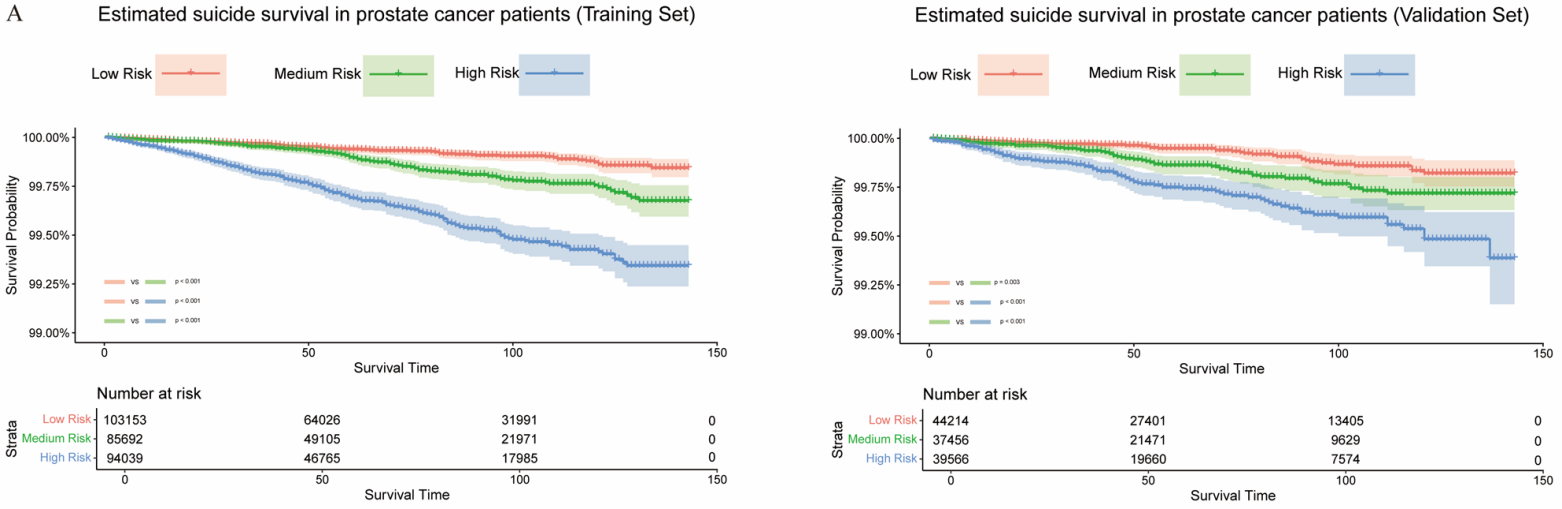
Kaplan-Meier curves of patients in the training set (**A**), validation set (**B**) according to risk grouping

We thoroughly assessed the predictive ability of the nomogram using the C-index and ROC curves. In the training set, the C-index was 0.714, and the AUCs for 1-year, 3-year, and 5-year survival were 0.7097, 0.7386, and 0.7334, respectively (Fig. 4B), demonstrating strong predictive performance. In the validation set, the C-index was 0.685, with AUCs of 0.6832 (1-year), 0.6829 (3-year), and 0.7006 (5-year) (Fig. 4C), further confirming the model’s robustness and excellent discrimination ability in an independent dataset, highlighting its strong external validation performance. However, univariate ROC analysis revealed that individual variables had limited predictive power (Figure S3), emphasizing the crucial role of multivariate analysis in enhancing accuracy. Moreover, the calibration curve (Fig. 5) illustrated the model’s good predictive accuracy. To refine suicide risk assessment in PCa patients, we categorized them into low, medium, and high-risk groups based on the tertiles of the risk score (351 and 385). Kaplan-Meier analysis showed significant survival differences between these risk groups (Fig. 6), further validating the model’s clinical utility.

## Discussion

Using data of 475,139 PCa patients from SEER database, we systematically analyzed baseline clinical characteristics and ranked causes of death. And we found an overall suicide rate of 25.4 per 100,000 person-years. This result was consistent with previous reports in other study [12]. Also, in a former study [22], high-risk PCa was linked to increased relative rates of depression and mortality. Taken together, the identification of higher suicide risk in older, unmarried, and non-urban patients, along with evidence that high-risk PCa is associated with depression and mortality, suggests that more mental health interventions and support are required in treatment. For better psychosocial distress detection and suicide intervention, LASSO regression and multivariable Cox proportional hazards models were used. We identified nine prognostic factors, including age, race, residence, tumor M stage, PSA level, Gleason score, surgical treatment, marital status, and radiotherapy, and developed a survival model based on these variables. To further assess the robustness of these risk factors in the overall cohort, we performed competing-risk analyses, given that many patients die from causes other than suicide. The results indicated that, except for radiotherapy, all other identified risk factors remained statistically significant, and stratified CIF curves further validated these associations. We interpret the diminished effect of radiotherapy as reflecting its protective role primarily among patients with longer survival, whereas this benefit may be obscured in the overall population due to competing risks. Nevertheless, considering radiotherapy is a standard treatment modality in prostate cancer and to ensure that our model remains clinically interpretable [23], we decided to retain it in the final model construction.

Overall, our study is consistent with previous researches of prostate cancer patients [24, 25]. However, most former studies focus on patients whose PSA levels were under 10 ng/mL [26, 27], while our study incorporating more high PSA level patients. The exclusion of such patients in earlier studies may introduce bias into predictive models, as elevated PSA levels are often associated with treatment failure or worse prognosis [28]. Additionally, we found that patients who did not undergo radical treatment or surgery exhibited a significantly higher suicide risk. Common reasons for not receiving surgery include refusal, financial constraints, and loss of surgical opportunity [29]. Patients who refuse surgery may be caught between concerns that not undergoing surgery will lead to disease progression and worries that surgery may impair postoperative quality of life and functional abilities. This conflict can lead to anxiety and depression, potentially increasing the risk of suicidal ideation [30, 31]. Therefore, clinical attention should be paid to the mental health of patients who refuse surgery, and appropriate psychosocial support should be provided. In contrast, patients who lose the opportunity for surgery and cannot undergo treatment often face poorer prognosis and uncontrollable disease progression, which may also exacerbate psychological stress and feelings of hopelessness, thereby increasing suicide risk.

Notably, Marilia et al. confirmed that PCa patients with anxiety, depression, or prior psychiatric treatment history face substantially greater suicide risk [32], warranting particular clinical attention. A study additionally identified prior depression history as a suicide risk factor in PCa patients [33], a variable not captured in our study, highlighting the need for expanded psychiatric comorbidity assessment in future research. Multiple recent studies highlight substantially increased suicide risk in PCa patients shortly after diagnosis [34, 35]. Aligning with our results, Sigrid et al. reported markedly elevated suicide risk within 6 months post-diagnosis [36]. We therefore recommend systematic mental health screening at PCa diagnosis, followed by prompt psychosocial support and interventions. Diagnosis age ≥ 80 years and unmarried status showed significant associations with increased suicide risk in PCa patients [12, 22, 37]. These factors represent key predictors of suicidal behavior in PCa patients, consistent with our analysis. Specifically, elderly patients often experience a decline in quality of life due to physiological deterioration and chronic diseases, which in turn increases the risk of mental health issues. Additionally, unmarried status is often associated with a lack of social support and feelings of loneliness, further exacerbating psychological burden and increasing suicide risk [38, 39].

This study constructed a large-scale cohort of 475,139 PCa patients based on the SEER database and successfully developed and validated a nomogram-based suicide risk prediction model. Compared with previous studies, this study is the first to include such a large patient population and systematically focus on the epidemiology of suicide risk among PCa patients over the past decade, providing new insights into this field. The model demonstrated excellent discriminatory ability and maintained stable predictive performance in the validation cohort. We believe that future application of this model in clinical practice could help promote the development of targeted psychological health screening programs, enable healthcare providers to identify high-risk prostate cancer patients earlier, and deliver timely mental health support, thereby improving overall patient outcomes.

However, this study has certain limitations. First, as a retrospective study, it relies on existing medical records, which may miss critical suicide-related factors such as a history of mental illness and prior suicide attempts. Second, the model may be influenced by competing risks and potential biases inherent in the dataset. As a result, the accuracy of suicide risk predictions could be affected, particularly when accounting for deaths from other causes and unmeasured confounding factors. Third, although endocrine therapies such as androgen deprivation therapy play a critical role in the management of prostate cancer, information on these treatments in the SEER database is incomplete and could not be included in our analysis. This omission may limit the comprehensiveness of the risk assessment, and future studies should incorporate more complete treatment records to further improve the accuracy and applicability of the model. Additionally, although the model has undergone internal validation, it has yet to be externally validated in independent cohorts, and differences in healthcare delivery, insurance coverage, and social support structures between the U.S. healthcare system and other settings may further limit the generalizability of our findings. Future studies incorporating multi-center data for external validation will be essential to assess the model’s generalizability and further optimize its clinical applicability.

## Conclusion

This study systematically evaluated mortality trends in prostate cancer patients over the past decade and identified key predictors of suicide. Building on these findings, we successfully developed a suicide risk prediction model, providing a precise tool for individualized risk assessment in clinical practice. This model facilitates early intervention and psychological support, aiming to reduce suicide risk among prostate cancer patients.

## Supplementary Information

Below is the link to the electronic supplementary material.

Supplementary Material 1

Supplementary Material 2

## Data Availability

The data used for analysis in this study are available in the supplementary materials. Further detailed information can be accessed from the SEER database (https://seer.cancer.gov) and the CDC Web-based Fatal Injury and Violence Reports (https://wisqars.cdc.gov/nvdrs).

## Abbreviations

AUC: Area Under the Curve
CDC: Centers for Disease Control and Prevention
Cl: Confidence interval
CIF: Cumulative incidence function
LASSO: Least Absolute Shrinkage and Selection Operator
HR: Hazard Ratio
PCa: Prostate Cancer
RP: Radical Prostatectomy
SEER: Surveillance, Epidemiology, and End Results
SD: Standard Deviations
sHR: Sub-distribution Hazard Ratio
SMR: Standardized Mortality Ratio
