# Supplementary material for: Suicide risk in prostate cancer patients: epidemiological trends and a predictive modeling
Source: BMC Psychiatry. 2026 Jan 19;26:163. doi: 10.1186/s12888-026-07806-7 (PMC12903677; doi:10.1186/s12888-026-07806-7)

**Table S1:** Suicide rates and SMRs among prostate cancer patients (2010–2021).

| **Variable** | **Suicide death** | **Pearson-year** | **Suicide rate per 100,000 person-years** | **SMR (95%Cl)** |
| --- | --- | --- | --- | --- |
| **Patients** | 625 | 2458414.1 | 25.4 | 1.198 (1.107-1.297) |
| **Diagnosis Year** |  |  |  |  |
| 2010-2013 | 361 | 1348183.5 | 26.8 | 1.321 (1.188 - 1.465) |
| 2014-2017 | 201 | 788456.3 | 25.5 | 1.188 (1.029 - 1.364) |
| 2018-2021 | 63 | 321774.2 | 19.6 | 0.89 (0.684 - 1.139) |
| **Age** |  |  |  |  |
| <60 | 165 | 611214.2 | 27 | 1.002 (0.855 - 1.167) |
| 60-69 | 231 | 1120733.1 | 20.6 | 0.793 (0.694 - 0.903) |
| 70-79 | 176 | 619574.8 | 28.4 | 0.989 (0.848 - 1.146) |
| >=80 | 53 | 106891.8 | 49.6 | 1.182 (0.885 - 1.546) |
| **Race** |  |  |  |  |
| Black | 38 | 377860.6 | 10.1 | 1.049 (0.742 - 1.439) |
| Other | 12 | 143070 | 8.4 | 0.716 (0.37 - 1.251) |
| White | 575 | 1937483.5 | 29.7 | 1.243 (1.144 - 1.349) |
| **PSA Level** |  |  |  |  |
| <=4 | 60 | 296100.3 | 20.3 | 0.954 (0.728 - 1.228) |
| >20 | 97 | 240821.9 | 40.3 | 1.896 (1.538 - 2.313) |
| 10-20 | 120 | 383362.2 | 31.3 | 1.474 (1.222 - 1.762) |
| 4-10 | 348 | 1538129.7 | 22.6 | 1.065 (0.956 - 1.183) |
| **Gleason Score** |  |  |  |  |
| Gleason Score<=6 | 210 | 1003366.2 | 20.9 | 0.985 (0.857 - 1.128) |
| Gleason Score=7 | 248 | 1004139.8 | 23.5 | 1.163 (1.023 - 1.317) |
| Gleason Score>=8 | 167 | 450908.1 | 37 | 1.744 (1.489 - 2.029) |
| **T Stage** |  |  |  |  |
| T0+TX | 13 | 28235.08 | 46 | 2.168 (1.154 - 3.707) |
| T1 | 283 | 1054793 | 26.8 | 1.263 (1.12 - 1.419) |
| T2 | 240 | 1047321.33 | 22.9 | 1.079 (0.947 - 1.224) |
| T3 | 79 | 311639.33 | 25.3 | 1.193 (0.945 - 1.487) |
| T4 | 10 | 16425.33 | 60.9 | 2.866 (1.375 - 5.271) |
| **N Stage** |  |  |  |  |
| N0+NX | 602 | 2382180.6 | 25.3 | 1.19 (1.097 - 1.289) |
| N1 | 23 | 76233.5 | 30.2 | 1.42 (0.9 - 2.131) |
| **M Stage** |  |  |  |  |
| M0 | 587 | 2394467.92 | 24.5 | 1.154 (1.063 - 1.251) |
| M1 | 38 | 63946.17 | 59.4 | 2.798 (1.98 - 3.84) |
| **Chemotherapy** |  |  |  |  |
| No/Unknown | 617 | 2440875.67 | 25.6 | 1.19 (1.098 - 1.288) |
| Yes | 8 | 17538.42 | 46 | 2.148 (0.927 - 4.232) |
| **Radiotherapy** |  |  |  |  |
| No/Unknown | 402 | 1573986.9 | 25.5 | 1.202 (1.088 - 1.326) |
| Yes | 223 | 884427.2 | 25.2 | 1.187 (1.036 - 1.354) |
| **Marital Status** |  |  |  |  |
| Married | 323 | 1657637.5 | 19.5 | 0.917 (0.82 - 1.023) |
| Other | 207 | 544938.2 | 38 | 1.788 (1.553 - 2.049) |
| Single | 95 | 255838.3 | 37.1 | 1.748 (1.414 - 2.137) |
| **Residence** |  |  |  |  |
| Metropolitan | 517 | 2174508.8 | 23.8 | 1.057 (0.968 - 1.152) |
| Non-metropolitan | 108 | 283905.2 | 38 | 1.691 (1.387 - 2.041) |
| **Median Household Income, $** |  |  |  |  |
| <45000$ | 23 | 88109 | 26.1 | 1.229 (0.779 - 1.844) |
| >=75000$ | 273 | 1115657 | 24.5 | 1.024 (0.907 - 1.153) |
| 45000$-74999$ | 329 | 1254648 | 26.2 | 1.388 (1.242 - 1.547) |
| **Surgical Treatment** |  |  |  |  |
| None | 411 | 1438486.2 | 28.6 | 1.345 (1.218 - 1.482) |
| Other | 38 | 105817.3 | 35.9 | 1.691 (1.196 - 2.321) |
| RP | 176 | 914110.5 | 19.3 | 0.906 (0.778 - 1.051) |

**Table S2:** Multivariable Cox regression and Fine-Gray competing risk regression analyses on suicide risk factors in prostate cancer patients.

| Variable | multivariate Cox regression analysis | | Fine-Gray analysis | |
| --- | --- | --- | --- | --- |
|  | HR (95%Cl) | P Value | sHR (95%Cl) | P Value |
| Age |  |  |  |  |
| <60 | Reference |  | Reference |  |
| 60-69 | 0.74 (0.58-0.95) | 0.018* | 0.66 (0.54-0.81) | <0.001*** |
| 70-79 | 0.96 (0.73-1.26) | 0.8 | 0.74 (0.59-0.93) | 0.0096** |
| >=80 | 1.88 (1.27-2.78) | 0.0016** | 1.29 (1.05-1.497) | 0.0032** |
| Race |  |  |  |  |
| Black | Reference |  | Reference |  |
| Other | 1.03 (0.48-2.20) | 0.94 | 1.03 (0.54-1.98) | 0.93 |
| White | 3.49 (2.36-5.16) | <0.001*** | 3.55 (2.54-4.97) | <0.001*** |
| Gleason Score |  |  |  |  |
| <=6 | Reference |  | Reference |  |
| 7 | 1.43 (1.13-1.82) | 0.0029** | 2.31 (1.04-1.54) | 0.021* |
| >=8 | 1.93 (1.44-2.59) | <0.001*** | 1.93 (1.44-2.59) | 0.0012** |
| T Stage |  |  |  |  |
| T0/Tx | Reference |  |  |  |
| T1 | 0.94 (0.46-1.92) | 0.86 |  |  |
| T2 | 0.92 (0.45-1.89) | 0.82 |  |  |
| T3 | 1.01 (0.47-2.19) | 0.98 |  |  |
| T4 | 1.47 (0.52-4.11) | 0.46 |  |  |
| M Stage |  |  |  |  |
| M0 | Reference |  | Reference |  |
| M1 | 1.77 (1.12-2.79) | 0.015* | 1.88 (1.21-2.31) | 0.0091** |
| Radiotherapy |  |  |  |  |
| No/Unknown | Reference |  | Reference |  |
| Yes | 0.84 (0.67-0.94) | 0.036* | 0.83 (0.69-1.21) | 0.072 |
| PSA Level |  |  |  |  |
| <=4 | Reference |  | Reference |  |
| 4-10 | 1.40 (0.97-2.01) | 0.071* | 1.14 (0.87-1.51) | 0.34 |
| 10-20 | 1.74 (1.15-2.61) | 0.008** | 1.38 (1.01-1.91) | 0.044* |
| >20 | 2.15 (1.38-3.33) | <0.001*** | 1.48 (1.12-1.82) | 0.0032** |
| Marital Status |  |  |  |  |
| Married | Reference |  | Reference |  |
| Other | 2.02 (1.64-2.49) | <0.001*** | 1.80 (1.51-2.15) | <0.001*** |
| Single | 1.93 (1.38-3.33) | <0.001*** | 1.95 (1.54-2.46) | <0.001*** |
| Median Household Income |  |  |  |  |
| <45000$ | Reference |  |  |  |
| 45000$-74999$ | 1.08 (0.66-1.77) | 0.75 |  |  |
| >=75000$ | 0.77 (0.46-1.30) | 0.33 |  |  |
| Surgery |  |  |  |  |
| None | Reference |  | Reference |  |
| Other | 1.08 (0.72-1.64) | 0.7 | 1.01 (0.72-1.42) | 0.95 |
| RP | 0.57 (0.42-0.78) | <0.001*** | 0.69 (0.55-0.86) | 0.0012** |
| Residence |  |  |  |  |
| Metropolitan | Reference |  | Reference |  |
| Non-metropolitan | 1.38 (1.04-1.82) | 0.025* | 1.43 (1.16-1.76) | <0.001*** |

HR = hazard ratio; sHR = sub-distribution hazard ratio; CI = confidence interval. *: 0.01<=P<0.05; **: 0.001<=P<0.01; ***: P<0.001

**Figure S1:** Cumulative incidence function (CIF) curves of suicide among patients with prostate cancer, stratified by significant risk factors. The curves depict the cumulative probability of suicide over time, with death from other causes treated as a competing event. Differences between groups were assessed using Gray’s test.


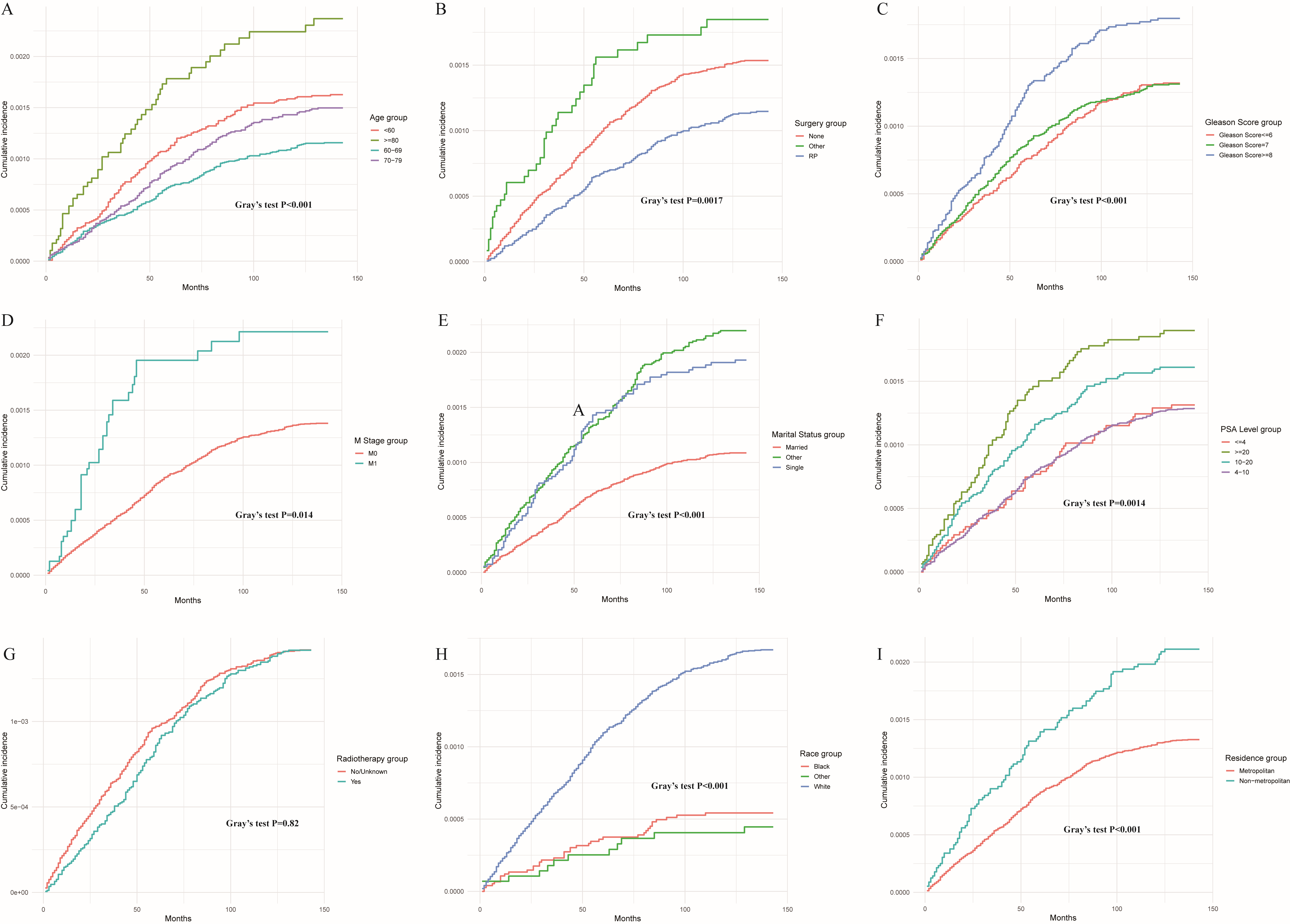


**Figure S2：**Time-varying Schoenfeld residuals plot.


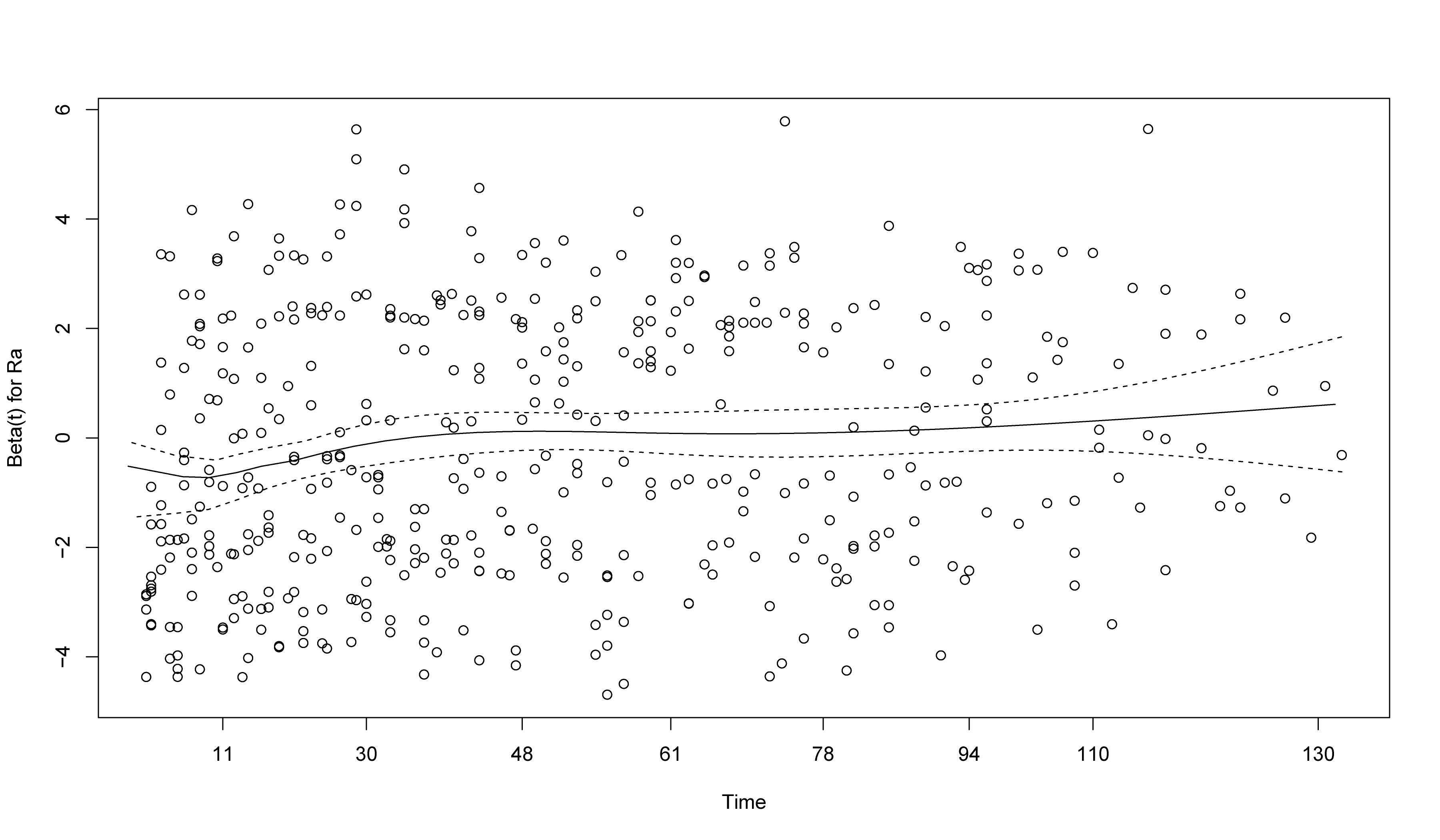


**Figure S3:** The ROC curve evaluates the predictive ability of a single factor, assessing its discriminative power for the target event.


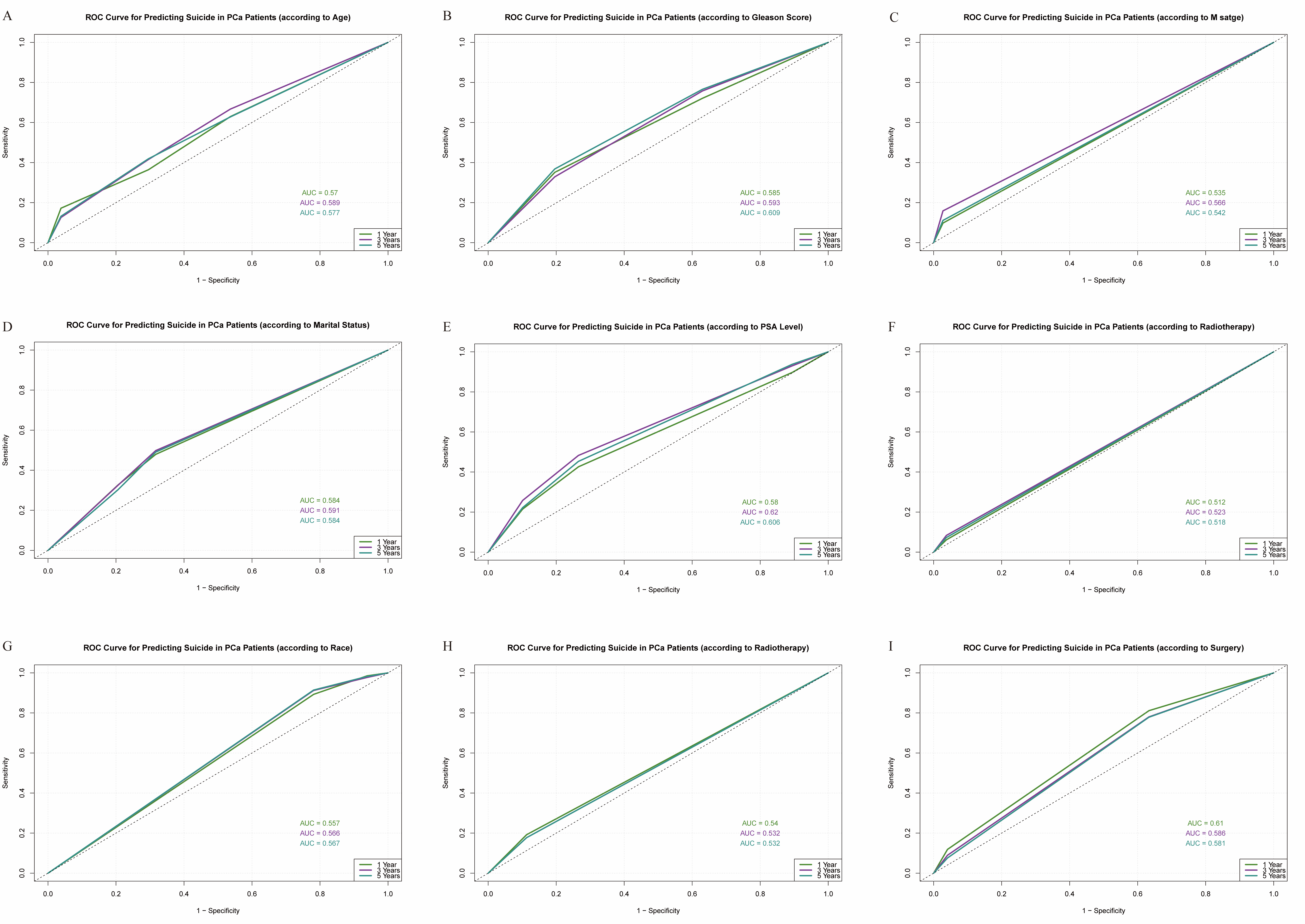

Supplement: Supplementary file 2 — Supplementary Material 2 [file 12888_2026_7806_MOESM2_ESM.docx]
